# Reviewers Acknowledgement

**DOI:** 10.1002/1878-0261.13581

**Published:** 2024-01-04

The Editors of *Molecular Oncology* would like to thank all referees who have given their time and expertise to review articles submitted for publication in Volume 17. *Molecular Oncology* heavily relies on reviewers' careful assessment, constructive criticism, and timely response to ensure both quality and fast turnaround times.

The names of these reviewers are listed below; we apologize if we have inadvertently omitted anyone.

Acebron Sergio

Agrawal Praveen

Ahmed Atique U

Ahmed Nuzhat

Aisner Dara L

Ajani Jaffer A

Akbari Abolfazl

Ali Ahmed

Allison Derek B

Almeida Raquel

Al‐Shaheri Fawaz N

Álvarez‐Calderón Iglesias Óscar

Alvarez‐Fernandez Monica

Alves Carla

Amat Ramon

Ambrogio Chiara

Amrutkar Manoj

Andersen Bogi

Angelastro James Michael

Anh Nguyen Hoang

Aniko Bozsik

Animesh Sambhavi

Antona Annamaria

Aran Veronica

Arcangeli Annarosa

Ariey‐Bonnet Jeremy

Arsenian‐Henriksson Marie

Arteaga Maria‐Francisca

Assoni Amanda F

Astanina Elena

Ávila Zaragozá Matías Antonio

Avril Tony

Babačić Haris

Babon Jeff J

Baiocchi Marta

Bajaj Jeevisha

Bakowsky Udo

Baldassarre Gustavo

Baltanas Fernando C

Bamias Aristotelis

Banerjee Sreeparna

Baniahmad Aria

Barbosa‐Morais Nuno

Barresi Valeria

Bartek Jiri

Barthet Valentin

Bartolomé Ruben A

Bartolomeo Sabrina Di

Bartoszewski Rafał

Bartsch Joerg Walter

Basu Sujit

Beird Hannah

Beltrán‐Anaya Fredy Omar

Benitez Riquelme Diego

Bennett Richard L

Berger Frédérique

Bergmann Michael

Bernasconi Michele

Bertrand Fred

Best Sarah

Bharadwaj Alamelu G

Bijlsma Maarten

Bilgin Mesut

Bird Tom

Biswas Antara

Bitler Benjamin G

Bjørge Line

Black Peter C

Blagih Julianna

Blandino Giovanni

Blanpain Cedric

Blum Yuna

Boccaccio Carla

Bochtler Tilmann

Boeri Mattia

Boini Krishna

Boncela Joanna

Bonora Elena

Bost Frederic

Boublikova Ludmila

Boussios Stergios

Braconi Chiara

Brancolini Claudio

Brest Patrick

Brinkmann Kerstin

Brizzi Maria Felice

Brodeur Mellica

Brooks Kelly

Brucoli Martina

Brummer Tilman

Brunner Cornelia

Buck Stefan

Bullova Petra

Bunse Lukas

Bunting Sam

Bussink Johan

Büttner Reinhard

Cabel Luc

Cai Zhixiong

Cai Yanyan

Callari Maurizio

Calzado Marco A

Campaner Stefano

Campbell Kristeen

Cani Andi

Cappello Paola

Caraglia Michele

Carbonell Caterina

Carrasco Alexis Germán Murillo

Carreras‐Puigvert Jordi

Carrier Alice

Carver Brett S

Casal J. Ignacio

Castaño Justo P

Catanzaro Giuseppina

Celetti Angela

Centanin Lazaro

Ceraline Jocelyn

Cerezo Michael

Chada Kiran

Chae Young Chan

Chaluvally‐Raghavan Pradeep

Chandra Joya

Chandrananda Dineika

Chang Wen‐Wei

Chang Wei‐Min

Chang Hong

Chapman Jessica

Chawlaa Ayesha

Chehelgerdi Mohammad

Chen BaoQing

Chen Li‐Han

Chen Jessica W

Chen Xin

Chen Ethan

Chen Qi

Cheng Binbin

Chern Edward

Cheung Lydia W T

Chin Wee

Choi Jieyon

Christopoulos Petros

Ciafré Silvia Anna

Ciardiello Fortunato

Cimini Daniela

Cives Mauro

Clynes Martin

Conteduca Vincenza

Cooke Mariana

Corà Davide

Corazzari Marco

Cordelier Pierre

Corman Alba

Corsetto Paola Antonia

Corujo David

Costa Bruno M

Coumans Frank

Courchet Julien

Courjaret Raphael

Cowling Victoria

Culig Zoran

Dabas Preeti

Dahlhoff Maik

Daidone Maria Grazia

Damia Giovanna

D'Angiolella Vincenzo

Darmoul Dalila

Daubon Thomas

Davidson Irwin

D'Avino Paolo

Davis Anthony

Dazert Eva

de Cárcer Guillermo

De Paula Bruno

De Smaele Enrico

de The Hugues

DeBono Johann

Del Sal Giannino

Deng Jinhai

Deng Wuguo

Derakhshan Fatemeh

Deshpande Ani

Deshpande Ravindra

Deuter Max

Di Fazio Pietro

Diaz Aaron

Díaz Caridad

Didonato Marzia

Dieguez Lorena

Dikoglu Esra

Dimas Konstantinos

Ding Ling‐Wen

Dirix Luc Y

Djouadi Fatima

Djouder Nabil

Donati Giulio

Dong Xuesen

Dopeso Higinio

Downs Jessica A

Dragomir Mihnea Paul

Drosten Matthias

Dubois Ludwig

Duncan James S

Duran Raul V

Eccles Michael

Echarri Asier

Edsjo Anders

Eferl Robert

Effenberger Katharina

Eide Inger Johanne

Elayat Ghada

Elkabets Moshe

Eriksson Pontus

Ernberg Ingemar

Espinet Elisa

Esposito Franca

Fahrmann Johannes F

Fang Wenfeng

Fang Yong

Farrell Paul

Feigin Michael

Fernández‐Zapico Martín

Ferraro Emanuela

Ferreri Carla

Ferretti Elisabetta

Filipska Martyna

Fischer Martin

Fisher Paul B

Flanagan Surangani Dharmawardhane

Fortunato Orazio

Francavilla Chiara

Friboulet Luc

Fu Jing

Furuya Naoki

Gammoh Noor

Ganapathy Kavya

Gandhi Minakshi

Gandhirajan Rajesh

Gao Ruize

Garayoa Mercedes

García de Herreros Antonio

Garcia Silva Susana

Gautier Mathieu

Gavard Julie

Gavathiotis Evripidis

Gebauer Niklas

Ghiringhelli Francois

Gil Valdes Jeovanis

Gires Olivier

Goel Ajay

Goldman Radoslav

Gorospe Myriam

Green Darrell

Gregor Martin

Greville Gordon

Grumati Paolo

Grusch Michael

Guan Haixia

Guan Chen

Guil Sonia

Guo Caixia

Gupta Avantika

Gupta Sanjay

Guren Marianne

Hackert Thilo

Haendler Bernard

Hagenfeld Daniel

Hamerlik Petra

Han Chuanhui

Hanash Samir M

Hannibal Luciana

Hao Jing

Harada Mamoru

Harada Hiroshi

Haricharan Svasti

Hatley Mark

He Bing

Henriksen Tenna Vesterman

Hernandez‐Valladares Maria

Hino Hirotsugu

Höglinger Carmen

Hsu Tsung‐I

Hu Shanshan

Huang Canhua

Hühn Daniela

Iczkowski Kenneth

Iglesias‐Ara Ainhoa

Ilangumaran Subburaj

Ilter Didem

Inderberg Else Marit

Iorio Marilena V

Iqbal Mohd Askandar

Issa Mark

Issaoui Hussein

Itoh Shinji

Ivanov Andrei

Ivanov Roman

Jalan Manisha

Jana Sujata

Janssen Klaus‐Peter

Jha Babal

Jiang Wei

Jiang Lei

Jin Wu Wen

Johannessen Bjarne

Johansson Mattias

Jouida Amina

Junker Kerstin

Kachhap Sushant K

Kamal Maud

Kanayama Mayuko

Kasimir‐Bauer Sabine

Kataoka Masako

Keeshan Karen

Keller Laura

Kenny Paraic

Kidron Heidi

Kim Sarah

Kim Alex

King Michael

Kiss‐Toth Endre

Kiuru Maija

Köbel Martin

Koc Emine

Kodous Ahmad

Koistinen Hannu

Kolch Walter

Kollmann Karoline

Kopetz Scott

Kortum Robert L

Krämer Oliver H

Kreuzaler Peter

Krueger Elke

Krzyscik Mateusz Adam

Kulasinghe Arutha

Kumar Ajay

Kumar Sushil

Kumari Sonam

Kung Hsing‐Jien

Kunnumakkara Ajaikumar Bahulayan

Kurelac Ivana

Kwong Joseph

Kzhyshkowska Julia

Lage Luís Alberto de Pádua Covas

Lal Ashish

Lalaoui Najoua

Lallemand Francois

Lallena Maria Jose

Landero‐Huerta Daniel

Latonen Leena

Lawrence Daniel

Lawrence‐Paul Matthew R

Lefebvre Tony Daniel

Lemarie Anthony

Lemecha Mengistu

León Javier

Leon‐Cabrera Sonia

Levanon Keren

Lewis Robert

Li Hui

Li Ming

Li Sha

Li Chen

Liang Tingming

Lianidou Evi

Liby Karen

Liefwalker Daniel F

Lim Seung‐Oe

Lin Zhen

Lindström Mikael

List Karin

Liu Ning‐Ning

Liu Jian

Liu Zeyi

Lofgren Kristopher

Lohr Matthias

Lokeshwar Bal L

Long Nguyen Phuoc

Long Jaclyn

Looijenga Leendert HJ

Lopez‐Contreras Andres Joaquin

Lord Christopher

Lowe Martin

Lu Yong‐Jie

Lum Julian J

Lumniczky Katalin

Luna Velez Maria Victoria

Luo Guanghong

Luo Bing

Luo Ji

Ma Jianping

Macip Salvador

Macurek Libor

Maes Brigitte

Maggiolini Marcello

Maksoud Semer

Malapelle Umberto

Malumbres Marcos

Manie Serge N

Marin‐Bejar Oskar

Marin‐Rubio Jose Luis

Marx Christian

Massey Andrew

Masson Glenn

Matei Daniela

Matias‐Guiu Xavier

Mauriello Alessandro

Maya‐Mendoza Apolinar

Mazumder Aloran

McEwan Iain

McEwan David

Mcilroy Marie

McIntyre Alan

Melo Sónia A

Menck Kerstin

Mereiter Stefan

Meza‐Zepeda Leonardo A

Miao Xiaoping

Michelena Jone

Michelucci Alessandro

Micke Patrick

Miele Evelina

Mieruszynski Stephen

Mikheev Andrei M

Mileshkin Linda

Milpied Pierre

Mineo Marco

Mitne‐Neto Miguel

Miyazono Kohei

Molina‐Vila Miguel A

Montero Joan

Montfort Anne

Montico Barbara

Montuenga Luis M

Moody Sarah C

Mora Jaume

Moreaux Jerome

Moreno Carlos

Mota Jorge

Moufarrij Sara

Mu Ping

Mueller Miryam

Mueller Volkmar

Muley Vilamu Helena

Muller Catherine

Muñoz Javier

Muratani Masafumi

Murphy Daniel James

Muthalagu Nathiya

Müther Michael

Nagy László

Nanjundan Meera

Napolitano Fabiana

Nasr Rihab

Nassar Farah

Nath Aritro

Nestal de Moraes Gabriela

Neubauer Hans

Neureiter Daniel

Nicchitta Christopher

Niehusmann Pitt

Nielsen Anders Lade

Niklison‐Chirou Maria Victoria

Nio Kouki

Nogués Laura

Nokin Marie‐Julie

Normanno Nicola

Notarangelo Michela

Nowak Dawid G

Ntwasa Monde

Oehme Ina

Ohta Satoshi

O'Neill Eric

O'Reilly Lorraine

Ortega‐Recalde Oscar

Oya Yuko

Pagano Michele

Pakala Suresh B

Palacios Jose

Palmer Ruth Helen

Palmieri Giuseppe

Palvimo Jorma J

Papandreou Ioanna

Parkes Eileen

Paschen Annette

Pasmant Eric

Pasquier Eddy

Pastuszak Krzysztof

Patel Areeba

Pei Xin

Pereira Patricia

Perez de Castro Ignacio

Perez‐Plasencia Carlos

Perren Aurel

Perry Rachel

Perucha Esperanza

Peschiaroli Angelo

Petermann Eva

Piaggio Giulia

Piekielko‐Witkowska Agnieszka

Pieraccioli Marco

Pierga Jean‐Yves

Piña‐Sanchez Patricia

Pirozzi Christopher James

Pisapia Pasquale

Po Agnese

Pol Jonathan

Pontis Francesca

Pors Klaus

Porta‐Pardo Eduard

Powell Michael Duane

Prekovic Stefan

Premkumar Daniel R

Price John T

Print Cris

Provenzani Alessandro

Pujadas Elisabet

Puri Raj K

Qvick Alvida

Radosa Julia

Radvanyi Francois

Rafei Hind

Raghunand Natarajan

Rainero Elena

Rajagopal Senthilkumar

Ramachandran Srinivas

Ranson Marie

Ratajewski Marcin

Rauch Jens

Ravi Arvind

Raynaud Florence

Rehman Fawad Ur

Rexer Brent N

Reynolds Jordan P

Rhee Sangmyung

Ribeiro Martha

Richmond Ann

Riediger Anja Lisa

Roberg Karin

Robledo Mercedes

Rocheleau Christian E

Rodrigues Pedro

Rodrigues Manuel

Rodriguez Galaxia Maria

Roesch Alexander

Rogatto Silvia Regina

Romano Maria Fiammetta

Rosell Rafael

Rotblat Barak

Ruess Dietrich A

Rye Morten B

Ryouhei Ohtani

Safonov Anton

Saha Biswarup

Sakunrangsit Nithidol

Sala Gianluca

Saluja Daman

Salvatore Domenico

Samuel Michael

Sanchez‐Burgos Laura

Sanchez‐Valle Jon

Sandulache Vlad C

Sangfelt Olle

Sanjay Kumar Singh

Santamaria David

Santamaria‐Martínez Albert

Santamarina‐Ojeda Pablo

Santhekadur Prasanna Kumar

Santocanale Corrado

Santoni‐Rugiu Eric

Saucier Caroline

Saur Dieter

Saw Stephanie

Scaffidi Paola

Scala Stefania

Scherer Florian

Schettini Francesco

Schilling Bastian

Schilling Oliver

Schlom Jeffrey

Schmitt Nicole C

Schmitz Ulf

Schneider Stock Regine

Schulz Wolfgang

Schuuring Ed

Schwaller Juerg

Scortegagna Marzia

Segers Rony

Seki Naohiko

Seoighe Cathal

Seymour Leonard

Shams Zinat

Sharma Aarti

Shen Sensi

Shen Jianzhong

Shen Lunda

Shi Hanping

Shi Jian

Sijts Alice JAM

Silvennoinen Olli

Simpson Daniel

Singh Ratnakar

Sinha Rohit A

Sirozh Oleksandra

Siska Peter

Sjödahl Gottfrid

Smirnov Artem

Smit Daniel

Smits Ron MJM

Soler Eric

Soller Matthias

Sonvico Fabio

Span Paul

Speirs Valerie

Spindler Karen‐Lise Garm

Srivastava Shiv

Stankevicius Vaidotas

Stark Jeremy

Steingrimsson Eirikur

Stites Edward

Stoecklein Nikolas H

Stoiber Dagmar

Strandgaard Trine

Strauss Robert

Sujobert Pierre

Sun Bin

Suzuki Rei

Syljuåsen Randi G

Szabo Roman

Tabernero Arantxa

Tagliabue Elda

Tait Stephen

Takahashi Shinichiro

Takayama Kenichi

Talbert Erin

Tanaka Yoshinori

Tanaka Hiroyoshi Y

Tang Hailin

Tang Daolin

Tasken Kristin

Tavassoli Mahvash

Tekpli Xavier

Terng Harn‐Jing

Testa Ugo

Thangarajah Fabinshy

Tiensuu Janson Eva

Timpson Pau

Toledo Rodrigo A

Toretsky Jeffrey A

Toth Reka

Touil Yasmine

Trader Darci

Tramm Trine

Treps Lucas

Trillsch Fabian

Tripathi Satyendra Chandra

Triulzi Tiziana

Trost Matthias

Tukachinsky Hanna

Turley Eva A

Udager Aaron M

Uguen Arnaud

Ulasov Ilya

Valinciute Gintvile

van den Bossche Jan

van den Broek Daan A

Vandeweyer Geert

Vanoli Fabio

VanSaun Michael N

Vasanthakumar Ajithkumar

Vecera Marek

Velasco Guillermo

Vergara Daniele

Vilchez David

Volpert Olga V

Volpi Emanuela

von Bubnoff Nikolas

von Felden Johann

Von Karstedt Silvia

Voutsadakis Ioannis A

Waldron Joseph

Wang Yonghua

Wang Yufeng

Wang Yu‐Shan

Watanabe Massayuki

Weber Thomas

Wei Wenyi

Weigert Andreas

Weiren Luo

Werner René

Westphal Manfred

Weyemi Urbain

Whitehurst Angelique

Whiteside Theresa L

Wiegering Armin

Wikman Harriet

Wilkins Anna

Winter Christof

Wong Kwok‐Chuen

Woods Nicholas

Worst Thomas S

Wozniak Agnieszka

Wu Min

Wu Dong‐Dong

Wu Kenneth Kun‐Y

Wurdak Heiko

Xiong Minmin

Xu Naihan

Xu Chuanrui

Xu Bei

Xu Dawei

Yamamoto Yusuke

Yang Haitao

Yang Xiaojun

Yin Shanshan

Yin Ling

Zhang Wenli

Zhang Xu Dong

Zhao Hongchang

Zhong Jun

Zhou Chumin

Zhu Hongwei

Zhu Ying

Zitzelsberger Horst

